# FKBP5 Mediates Alveolar Fibroblast Necroptosis During Acute Respiratory Distress Syndrome

**DOI:** 10.1111/cpr.70075

**Published:** 2025-06-17

**Authors:** Dong Zhang, Wei Liu, Ting Sun, Yangyang Xiao, Qiuwen Chen, Xiao Huang, Xiaozhi Wang, Qian Qi, Hao Wang, Tao Wang

**Affiliations:** ^1^ Department of Intensive Care Unit Binzhou Medical University Hospital Binzhou Shandong China; ^2^ Department of Clinical Laboratory Binzhou Medical University Hospital Binzhou Shandong China; ^3^ Department of Medical Technology Binzhou Polytechnic Binzhou Shandong China; ^4^ Department of Respiratory and Critical Care Medicine The First Affiliated Hospital of Shandong First Medical University and Shandong Provincial Qianfoshan Hospital, Shandong Institute of Respiratory Diseases Jinan China; ^5^ Department of Critical Care Medicine Qilu Hospital of Shandong University Jinan Shandong China

**Keywords:** acute respiratory distress syndrome, alveolar fibroblast, inflammatory storm, necroptosis

## Abstract

The inflammatory storm is a hallmark of acute respiratory distress syndrome (ARDS), yet effective therapies remain unavailable. FK506‐binding protein 51 (FKBP5) has emerged as a regulator of inflammatory responses. In this study, FKBP5 expression was markedly increased in patients with sepsis and correlated with both cytokine levels and disease severity. Using sepsis‐induced ARDS models in *Fkbp5* ^*−/−*^ and bone marrow chimeric mice, this study demonstrated that non‐haematopoietic FKBP5 mitigates inflammatory injury. Single‐cell transcriptomic analysis identified fibroblasts and epithelial cells as the primary sources of non‐haematopoietic FKBP5 in the lung injury. Conditional deletion of FKBP5 in fibroblasts (*Col1a2*‐iCre *Fkbp5* ^flox/flox^) confirmed the essential role of fibroblast FKBP5 in the inflammatory response during ARDS. Mechanistically, FKBP5‐mediated necroptosis of alveolar fibroblasts triggered NF‐κB activation, proinflammatory cytokine release, neutrophil recruitment, and the establishment of an inflammatory microenvironment in alveolar epithelial tissue. These findings suggest a potential therapeutic strategy targeting fibroblast FKBP5 and provide a foundation for future clinical investigation in ARDS management.

## Graphical abstract

Structure diagram of alveolar fibroblast activation in septic ARDS model. FKBP5‐mediated alveolar fibroblast necroptosis, leading to the activation of NF‐κB signalling and the secretion of cytokines and the recruitment of neutrophils, and further orchestrating the inflammatory environment of the alveolar epithelia in septic ARDS.

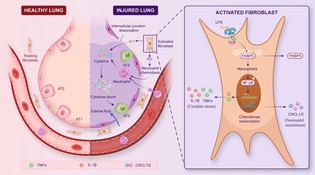

## 1 Introduction

Acute respiratory distress syndrome (ARDS) is a prevalent and severe condition that poses a significant threat to human health. Epidemiological studies indicate that ARDS patients constitute approximately 10% of all individuals hospitalised in intensive care units, with a mortality rate ranging from 30% to 50% [1]. ARDS represents a critical public health challenge that necessitates immediate attention and intervention [2]. The aetiology of ARDS is diverse, with sepsis identified as the primary pathogenic factor, accounting for about 30% of cases, whereas effective therapeutic options remain limited [3]. Although the role of the inflammatory response in ARDS is recognised, its regulatory mechanisms are not yet fully understood [4].

The inflammatory response denotes an exaggerated release of cytokines, including interleukins and chemokines, triggered by dysregulation of immune regulatory mechanisms. This phenomenon is closely associated with severe hyperinflammatory conditions, such as sepsis [5]. Lipopolysaccharide (LPS) serves as a significant contributor to septic ARDS, acting as a substantial exogenous trigger for inflammatory responses, leading to elevated levels of IL‐1β, TNF‐α, and procalcitonin (PCT) [6]. During the course of ARDS, there is recruitment of neutrophils and an excessive release of neutrophil extracellular traps (NETs). NETs can bind to endothelial or epithelial cells through histone modification, potentially inducing intravascular thrombosis, disrupting the alveolar epithelial barrier, and causing various forms of injury [7, 8]. The inflammatory response is a prominent feature of severe and critical ARDS and is viewed as a significant factor contributing to the high mortality associated with this condition [9, 10]. Currently, clinical treatment strategies for ARDS primarily focus on neutralising or inhibiting cytokines [11]. However, this approach lacks clarity regarding the precise sources of cytokines and suffers from a lack of specificity in therapeutic strategies.

In addition to their well‐established roles in tissue repair and fibrotic diseases, fibroblasts play a vital role in regulating the immune microenvironment. Fibroblasts are activated by various intracellular and extracellular signals, including cytokines, which enable them to initiate inflammation, participate in inflammatory responses, and modulate immune cell activity [12]. Research indicates that lung fibroblasts coordinate the progression and resolution of acute pulmonary inflammatory cascades through multiple mechanisms [13]. Furthermore, fibroblasts exhibit heterogeneity, with various subtypes displaying different functions [14]. A recent study demonstrated that Scube2 specifically marks alveolar fibroblasts, which modulate bleomycin‐induced lung inflammation [15]. Thus, the role of alveolar fibroblasts in inflammatory responses in ARDS remains unclear, as do the specific pathways.

Necroptosis is a significant trigger of the inflammatory response, mediated by receptor‐interacting protein kinases 1 (RIPK1), RIPK3, and mixed lineage kinase domain‐like protein (MLKL) [16]. Studies have shown that necroptosis inhibitors alleviate pulmonary injury by modulating the function of neutrophils or macrophages in LPS‐induced ARDS [17, 18]. Notably, RIPK1 and RIPK3 are necessary for MLKL activation in macrophages, whereas RIPK1 is not required in fibroblasts [19]. Furthermore, Zhang et al. demonstrated that inhibiting RIPK3/MLKL‐mediated necroptosis in gum fibroblasts alleviated periodontitis [20], suggesting that necroptosis of alveolar fibroblasts may drive cytokine storms and exacerbate inflammation in the ARDS process.

FK506 binding protein 51 (FKBP51, also known as FKBP5) is a member of the immunophilin class of proteins and is encoded by the FKBP5 gene located on the short arm of chromosome 6 [21]. Research indicates that FKBP5 plays a critical role in the inflammatory response and immune regulation by modulating various signalling pathways [22, 23]. Inhibition of FKBP5 has demonstrated certain anti‐inflammatory effects, highlighting its potential as a therapeutic target for inflammation‐related diseases.

This study developed *Fkbp5* ^*−/−*^ mice, bone marrow chimeric mice, and *Col1a2*‐iCre *Fkbp5* ^flox/flox^ mice to further investigate how FKBP5‐mediated necroptosis of alveolar fibroblasts orchestrates the inflammatory environment in ARDS. The findings from this research will enhance the understanding of new pathogenic mechanisms underlying septic ARDS, as well as facilitate the identification and development of therapeutic targets, drugs, and novel diagnostic and treatment strategies for the prevention and management of septic ARDS, ultimately benefiting patients.

## 2 Methods and Materials

### 2.1 Reagents

Small interfering RNA (siRNA) negative control (NC) and siRNA *Fkbp5* were synthesised by General Biology (Anhui, China). LPS (O55:B5), 4′,6‐diamidino‐2‐phenylindole dihydrochloride (DAPI), and fluorescein isothiocyanate‐dextran (FITC‐dextran, 60842‐46‐8) were purchased from Sigma‐Aldrich (Louis, USA). The following antibodies were obtained from HUABIO (Zhejiang, China): anti‐occludin (R1510‐33), anti‐HMGB1 (ET1601‐2), anti‐IL‐6 (R1412‐2), anti‐TNF‐α (ER1919‐22), anti‐NF‐κB P65 (ET1603‐12), anti‐phospho‐NF‐κB P65 (S536, HA723223), anti‐IκBα (ET1603‐6), anti‐phospho‐IκBα (S32, ET1609‐78), anti‐RIPK3 (ER1901‐27), anti‐PAD4 (HA721657), anti‐syndecan‐1 (SDC‐1, ET1703‐42), anti‐GAPDH (ET1601‐4), and HRP‐conjugated goat anti‐rabbit IgG (HA1001). Anti‐MLKL (RM8381) and anti‐RIPK1 (BD‐PN1850) were sourced from Biodragon (Suzhou, China). Anti‐heparan sulphate proteoglycan (HS) was obtained from Millipore (Temecula, USA). The BCA protein assay kit (PC0020), haematoxylin–eosin (H&E) staining kit (G1120), and anti‐ZO‐1 (K001718P) were supplied by Solarbio (Beijing, China). Anti‐scube2 (PA5‐20991) and Opti‐MEM were purchased from Thermo Fisher Scientific (Waltham, USA). NanoTrans transfection reagent for siRNA (CT0003) was obtained from CYTOCH (Shanghai, China). Additional antibodies—including anti‐scube2 (PC12318), anti‐human phospho‐RIPK3 (Ser316, TA4508), and anti‐mouse phospho‐RIPK3 (Ser232, TA7443)—were obtained from Abmart (Shanghai, China). Human alveolar fibroblasts (ZQY032) and type II alveolar epithelial cells (ZQY095) were purchased from Zhong Qiao Xin Zhou Biotechnology (Shanghai, China). The cell counting kit‐8 (C0037) and necroptosis inducer kit containing TSZ (C1058) were obtained from Beyotime Biotechnology (Shanghai, China). Anti‐phospho‐MLKL (Ser358, AF7420), anti‐phospho‐RIPK1 (Ser166, AF2398), anti‐FKBP5 (DF13305), and anti‐CXCL2 (DF12551) were sourced from Affinity Biosciences (Jiangsu, China). Anti‐histone H3 (citrulline R2 + R8 + R17, CiH3, ab5103) was purchased from Abcam (Cambridge, UK). Anti‐Ly6G (GB11229) was obtained from Servicebio Biosciences (Wuhan, China). Anti‐MPO (22225‐1‐AP) and anti‐collagen I (66761‐1‐Ig) were sourced from Proteintech (Wuhan, China). The Quant‐iT PicoGreen dsDNA assay kit (P11496) was obtained from Invitrogen (Carlsbad, USA). The ELISA kit for FKBP5 (SEE645) was purchased from Cloud‐Clone Corp. (Wuhan, China). ELISA kits for TNF‐α (EK182, EK282), IL‐1β (EK101B, EK201B), IL‐6 (EK206), CXCL1 (EK196, EK296), and CXCL2 (EK1264, EK214) were sourced from Multi Sciences Biotech (Hangzhou, China). An ELISA kit for MPO (JL10367) was purchased from Jianglai Biology (Shanghai, China).

### 2.2 Bioinformatic Analysis of FKBP5 Based on Gene Expression Omnibus (GEO) Datasets

Gene expression datasets related to FKBP5 in sepsis‐derived whole blood were obtained from the GEO database (https://www.ncbi.nlm.nih.gov/geo/), including GSE13904, GSE26378, GSE63042, and GSE185263.

### 2.3 Human Subjects

This study was conducted at the Department of Intensive Care Unit, Binzhou Medical University Hospital, from January 2022 to July 2024 and registered under ChiCTR2100054516. Sepsis was diagnosed based on Sepsis 3.0 criteria. Inclusion criteria included patients aged 18 to 80 years within 24 h of admission. Exclusion criteria included age < 18 or > 80 years; confirmed COVID‐19 or influenza infection; autoimmune diseases; malignancy; chronic lung disease; long‐term glucocorticoid therapy; pregnancy; and heart failure or cardiac arrest. The study protocol was approved by the Human Research Ethics Committee of Binzhou Medical University Hospital. Healthy volunteers were recruited from the hospital's physical examination center. Peripheral blood (3–5 mL) was collected within 24 h of admission, and serum was stored at *−*80°C.

### 2.4 Construction Gene Mice

*Fkbp5* ^*−/−*^, *Fkbp5* ^flox/flox^, and *Col1a2*‐iCre *Fkbp5* ^flox/flox^ mice (C57BL/6, 6–8 weeks old) were obtained from Saiye Biology and housed in a specific‐pathogen‐free facility at Binzhou Medical University Hospital. All procedures were approved by the Institutional Animal Care and Use Committee. Sepsis ARDS was induced by intraperitoneal injection (i.p.) of LPS (20 mg/kg) for 6 h, as previously reported [24].

### 2.5 BM Chimeric Mice

Recipient wild‐type (WT) or *Fkbp5* ^*−/−*^ mice were irradiated with two doses of X‐rays (4.5 Gy each) at a 3‐h interval [25]. Donor WT or *Fkbp5* ^*−/−*^ bone marrow cells were harvested aseptically from the femur and tibia, and red blood cells were removed. A total of 1–2 × 10^6^ cells were injected via the tail vein. Four chimeric groups were generated: WT → WT, *Fkbp5* ^−/−^ → WT, WT → *Fkbp5* ^*−/−*^, and *Fkbp5* ^−/−^ → *Fkbp5* ^−/−^. Chimeric mice received sterile water with neomycin (0.5 mg/mL) to prevent infection. Immune reconstitution was confirmed after 8 weeks, followed by LPS challenge (20 mg/kg) for 6 h to induce ARDS.

### 2.6 Micro‐CT Imaging

Mice were anaesthetised with 1% pentobarbital sodium, and chest scans were performed using a micro‐CT imaging system (PerkinElmer‐Calliper LS, Boston, USA) [26].

### 2.7 Lung Permeability

Pulmonary vascular permeability was assessed using FITC‐dextran. Mice were injected via the tail vein with 100 μL FITC‐dextran (25 mg/mL), followed by PBS perfusion to clear circulating dye over 1 h. Fluorescence distribution in the lungs was captured using the AniView100 Pro imaging system.

### 2.8 Histopathology and Histological Analysis

Lungs were fixed in 4% paraformaldehyde, dehydrated in a graded ethanol series, and dewaxed with xylene. Samples were embedded in paraffin and sectioned at 5 μm for H&E staining. Lung injury was assessed using a standardised pathological scoring system [26].

### 2.9 Collection Bronchoalveolar Lavage Fluid (BALF)

A 1 mL syringe of cold PBS was inserted into the upper bronchial bifurcation, and the lungs were gently rinsed multiple times. BALF was centrifuged at 1000 rpm for 10 min at 4℃, and the supernatant was collected for analysis.

### 2.10 Flow Cytometry Analysis

BALF cell suspensions were stained with fluorophore‐conjugated anti‐Ly6G (BD Biosciences, 560602) and anti‐CD11b (BD Biosciences, 553310) antibodies at 4℃ for 30 min. Samples were analysed using a CytoFLEX flow cytometer, and data were processed with CytExpert software.

### 2.11 Enzyme‐Linked Immunosorbent Assay (ELISA)

Concentrations of FKBP5, cell‐free DNA (cfDNA), MPO, IL‐1β, TNF‐α, IL‐6, CXCL1, and CXCL2 in serum or BALF were quantified using commercial ELISA kits, following the manufacturers' protocols.

### 2.12 Pulmonary Ultrastructure

Mice received pulmonary perfusion with a fixative containing 2% glutaraldehyde, 2% sucrose, 0.1 M sodium cacodylate buffer, and 2% lanthanum nitrate. Lung tissue was rinsed with sodium cacodylate buffer, post‐fixed with 2% osmium tetroxide and 2% lanthanum nitrate, and examined by transmission electron microscopy (TEM) [27].

### 2.13 Immunofluorescence Analysis

Cells were seeded in 24‐well plates and incubated with primary antibodies (anti‐scube2, anti‐FKBP5, anti‐phospho‐MLKL, anti‐phospho‐RIPK1, and anti‐phospho‐RIPK3) for 12 h after treatment. After counterstaining with DAPI and incubation with fluorescent secondary antibodies, images were acquired by fluorescence microscopy. Lung tissues were processed (dehydration, dewaxing, antigen retrieval), stained with primary antibodies (anti‐PAD4, anti‐CiH3, anti‐MPO, anti‐collagen I, anti‐FKBP5, and anti‐scube2), and visualised with DAPI and fluorescent secondaries.

### 2.14 Immunoblotting

Proteins from cells or lung tissue were extracted, separated via SDS‐PAGE, and transferred to PVDF membranes. Membranes were blocked and incubated at 4 °C overnight with primary antibodies (CXCL2, HMGB1, IL‐1β, TNF‐α, PAD4, ZO‐1, occludin, HS, FKBP5, P65, phospho‐P65, IκBα, phospho‐IκBα, MLKL, phospho‐MLKL, RIPK1, phospho‐RIPK1, RIPK3, phospho‐RIPK3, and GAPDH). After washing with TBST, appropriate HRP‐conjugated secondary antibodies were applied for 1.5 h. Protein bands were visualised using chemiluminescent detection.

### 2.15 RNA Sequencing

Total RNA extraction, library construction, and sequencing were performed as previously described [28]. RNA‐seq was conducted by Shanghai Bioprofile Technology Co. Ltd., and bioinformatics analysis followed standard protocols.

### 2.16 Single Cell RNA‐Sequencing

Single‐cell RNA sequencing and associated analyses were conducted by Biomarker Technologies Corporation. Cell‐type identification markers included: epithelial cells (Epcam); endothelial cells (Pecam1, Lyve1, Vwf, Flt1, Chd5, Kdr, Cldn5, Ramp2); B cells (Cd19, Cd79a, Cd79b, Ms4a1, Igha, Igkc, Jchain); granulocytes (S100a9, S100a8, Ccl12); T cells (CD3D, CD3E, CD3G, CD8, Ccr7, CD3D, Icos, Ctla4, Tnfrsf4, CD3G, CD2); fibroblasts (Ccl6, Fabp4, Fabp1, Lpl, Mgp, Gsn, Col1a1); macrophages (CCl22, CCR7); and red blood cells (Hbb‐bs, Hba‐a2, Hba‐bt).

### 2.17 Mouse Alveolar Fibroblasts Isolation, Culture, and Treatment

The separation method follows the steps reported in previous studies [29]. Following anaesthesia, mice were wiped with 75% ethanol, and lung tissue was harvested under sterile conditions. Tissue was digested with type I collagenase and 0.25% trypsin at 37°C for 30 min. The supernatant was centrifuged, digestion was stopped, and cells were resuspended in DMEM. After red blood cell lysis, cells were filtered through a 200‐mesh strainer and seeded into T‐25 flasks. After 2 h of adherence, cells were washed with PBS containing antibiotics, and fresh medium was added. Spindle‐shaped fibroblasts were identified under microscopy. After 10 days, fibrocytes exhibited radial or palisade arrangements. Over 95% purity was confirmed by anti‐scube2 immunofluorescence. Cells were stimulated with LPS (10 μg/mL) for downstream experiments.

### 2.18 Human Alveolar Fibroblasts Culture and Treatment

Cells were seeded in 6‐well plates. On the following day, 1 μL NanoTrans was mixed with 50 μL Opti‐MEM; 20 pmol siRNA was diluted in another 50 μL Opti‐MEM. The two solutions were combined and incubated at room temperature for 20 min, then added to wells. Cells were incubated at 37℃ in 5% CO_2_ for 48 h. Following transfection, fibroblasts were treated with LPS (10 μg/mL) or TSZ (1:1000) for subsequent assays.

### 2.19 Transwell Coculture

Alveolar fibroblasts were seeded in the lower chambers of transwell inserts, and type II alveolar epithelial cells were seeded in the upper chambers. Fibroblasts were transfected with siRNA *Fkbp5* or siRNA NC and then stimulated with LPS (10 μg/mL). After 6 h of co‐culture, epithelial cells were harvested for analysis of glycocalyx and tight junction protein expression.

### 2.20 Statistical Analysis

Data are expressed as means ± standard deviations. Analyses were conducted using GraphPad Prism 10.0. Comparisons between two groups were performed using the unpaired *t*‐test (for normally distributed data) or the Mann–Whitney *U* test (for non‐normal data). For multiple group comparisons, one‐way ANOVA or Welch ANOVA was used for normally distributed data, and the Kruskal–Wallis test was used for non‐normally distributed data. Statistical significance was defined as *p* < 0.05.

## 3 Results

### 3.1 The Clinical Role of FKBP5 in Diagnosing Sepsis and Predicting Severity

**FIGURE 1:**
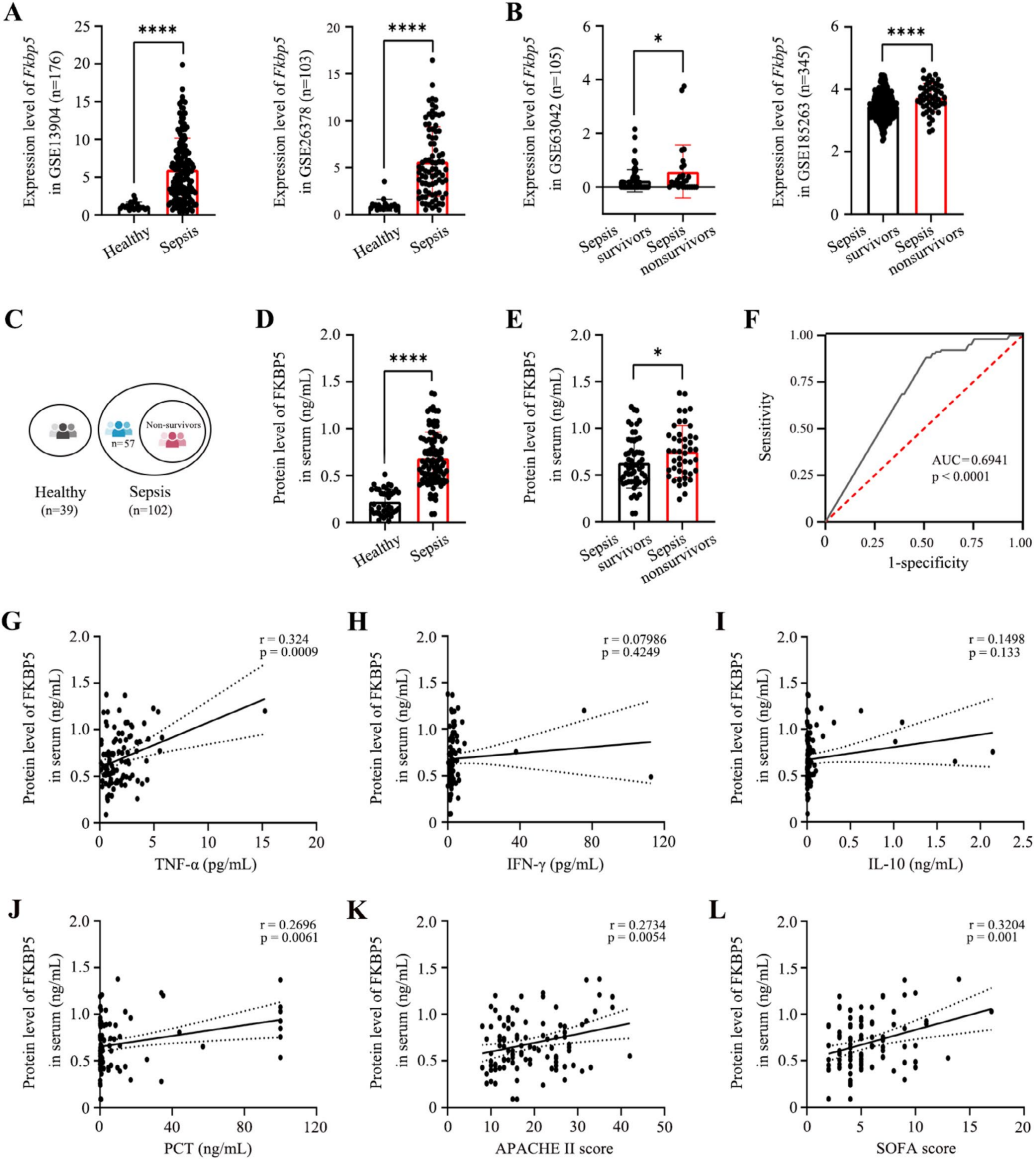
Clinical role of FKBP5 in diagnosing sepsis and predicting severity. (A) Transcriptome expression profiling of whole blood‐derived RNA from sepsis patients and healthy controls based on GEO datasets. (B) GEO datasets comparing *Fkbp5* level in survivors and non‐survivors with sepsis. (C) Schematic diagram of the study cohort: Healthy individuals (*n* = 39), survivors with sepsis (*n* = 57), and non‐survivors with sepsis (*n* = 45). (D) Plasma FKBP5 levels in sepsis patients and healthy volunteers. (E) Comparison of FKBP5 plasma levels between survivors and non‐survivors. (F) ROC curve analysis of FKBP5 for sepsis diagnosis. (G–L) Correlation analysis between serum FKBP5 and TNF‐α, IFN‐γ, IL‐10, PCT, APACHE II score, and SOFA score. All data are presented as means ± SD from three independent experiments. *p < 0.05, **p < 0.01, ***p < 0.001, ****p < 0.0001; ns, not significant.

Transcriptome expression profiling from GEO datasets was used to analyse RNA levels of FKBP5 in whole blood‐derived RNA from patients with sepsis and healthy controls. Bioinformatics analysis indicated that *Fkbp5* mRNA was significantly upregulated in sepsis patients compared to controls (Figure 1A) and showed a strong association with patient survival (Figure 1B). A total of 102 patients with sepsis and 39 healthy volunteers were recruited (Figure 1C). FKBP5 protein levels were markedly higher in sepsis patients than in healthy volunteers and significantly lower in survivors than in non‐survivors (Figure 1D,E). Receiver operating characteristic (ROC) curve analysis yielded an area under the curve (AUC) of 0.6941 (95% CI: 0.6332–0.7550, *p* < 0.0001) for FKBP5 in identifying sepsis (Figure 1F). Correlation analysis further demonstrated that FKBP5 levels positively correlated with TNF‐α (95% CI: 0.1383–0.4878, *p* = 0.0009), PCT (95% CI: 0.07925–0.4409, *p* = 0.0061), SOFA score (95% CI: 0.1343–0.4847, *p* = 0.001), and APACHE II score (95% CI: 0.08341–0.4443, *p* = 0.0054). However, no significant correlation was observed with IFN‐γ (95% CI: −0.1164–0.2701, *p* = 0.4249) or IL‐10 (95% CI: −0.04604–0.3345, *p* = 0.133) (Figure 1G–L). These findings indicate that FKBP5 is closely associated with both systemic inflammation and disease severity in sepsis.

### 3.2 FKBP5 Knockout Relieves the Development of Inflammation With Septic ARDS

**FIGURE 2:**
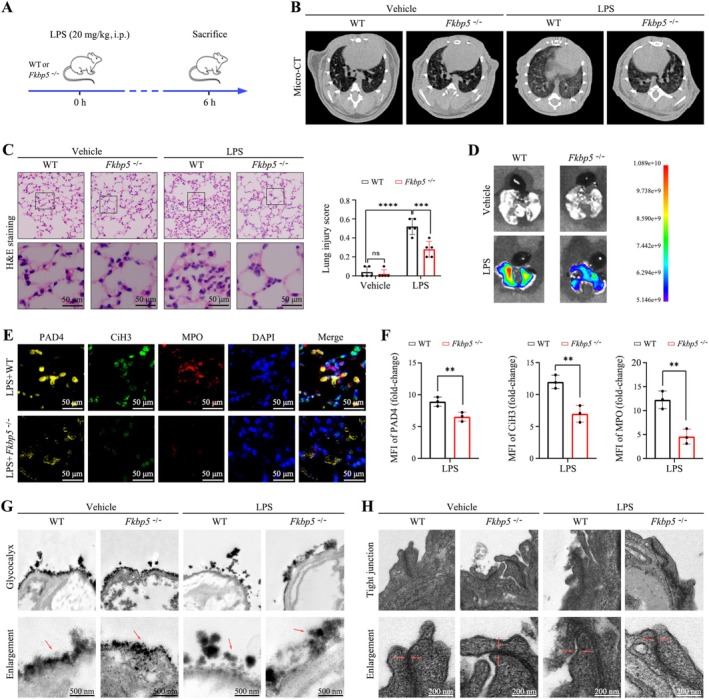
FKBP5 knockout alleviates inflammation in septic ARDS (*n* = 6). (A) Schematic of septic ARDS model in *Fkbp5* ^*−/−*^ mice. (B) Representative micro‐CT images of mouse lungs. (C) H&E staining and histological scoring (200×; scale bar, 50 μm). (D) FITC‐dextran imaging to assess vascular leakage. (E, F) Immunofluorescence images and quantification of PAD4, CiH3 and MPO in lung tissue (200×; scale bar, 50 μm). (G) Glycocalyx microstructure (8000×; scale bar, 500 nm). (H) Tight junction ultrastructure (20,000×; scale bar, 200 nm). All data are presented as means ± SD from three independent experiments. *p < 0.05, **p < 0.01, ***p < 0.001, ****p < 0.0001; ns, not significant.

A septic ARDS model was established using *Fkbp5* ^*−/−*^ mice to examine the function of FKBP5 in ARDS pathogenesis (Figure 2A). Micro‐CT imaging showed that *Fkbp5* ^*−/−*^ mice displayed no spontaneous lung lesions but had markedly reduced LPS‐induced pulmonary exudation (Figure 2B). Histopathological analysis confirmed that LPS caused alveolar structural disruption and inflammatory cell infiltration, which were significantly mitigated in *Fkbp5* ^*−/−*^ mice (Figure 2C). FITC‐dextran leakage assays further demonstrated that FKBP5 deficiency conferred protection against LPS‐induced pulmonary vascular permeability (Figure 2D). Immunofluorescence staining showed reduced levels of NETs markers (PAD4, CiH3, and MPO) in *Fkbp5* ^*−/−*^ mice compared to WT mice following LPS exposure (Figure 2E,F). TEM revealed that FKBP5 knockout protected alveolar epithelial cells from LPS‐induced glycocalyx degradation and tight junction disruption (Figure 2G,H). These results indicate that FKBP5 contributes to pulmonary inflammatory injury in septic ARDS.

### 3.3 FKBP5 Deficiency in Nonhematopoietic Cells Served as the Major Contributor to the Mitigation of Septic ARDS

**FIGURE 3:**
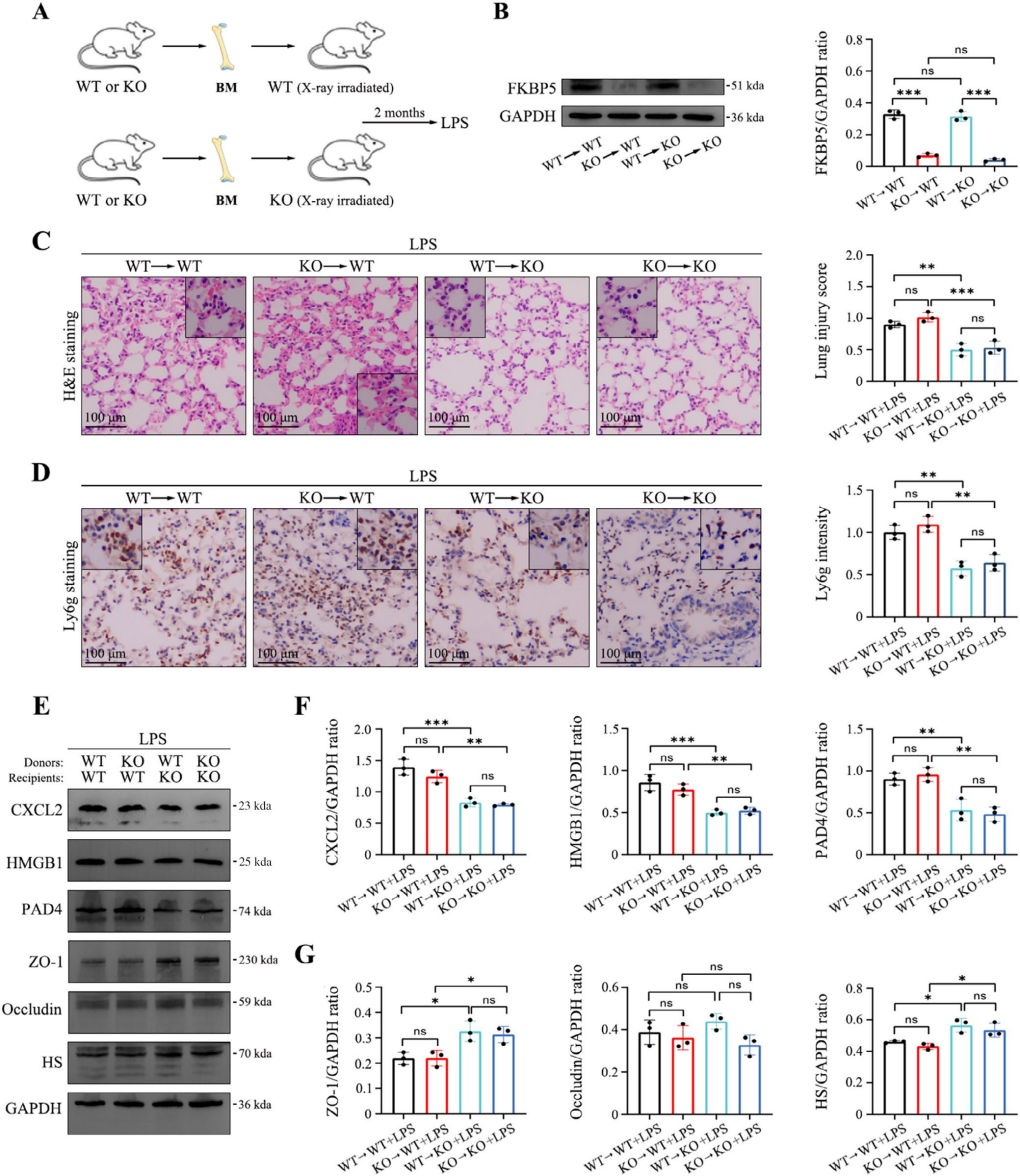
FKBP5 deficiency in non‐haematopoietic cells contributes to attenuation of septic ARDS. (A) Schematic of bone marrow transplantation protocol. (B) Confirmation of FKBP5 clearance efficiency in bone marrow cells. (C) H&E staining to assess inflammatory injury (100×; scale bar, 100 μm). (D) Ly6G immunostaining to evaluate neutrophil infiltration (100×; scale bar, 100 μm). (E–G) Immunoblots and quantification of glycocalyx components, tight junction proteins, and inflammatory cytokines. All data are presented as means ± SD from three independent experiments. *p < 0.05, **p < 0.01, ***p < 0.001, ****p < 0.0001; ns, not significant.

To distinguish the role of FKBP5 in haematopoietic versus non‐haematopoietic cells, reciprocal bone marrow transplantations were performed to generate four groups of chimeric mice: WT → WT, *Fkbp5* ^−/−^ → WT, WT → *Fkbp5* ^−/−^, and *Fkbp5* ^−/−^ → *Fkbp5* ^−/−^ (Figure 3A,B). Analysis of pulmonary pathology indicated that FKBP5 deficiency in non‐haematopoietic cells significantly reduced lung injury, highlighting their predominant role in regulating ARDS severity (Figure 3C–G).

**FIGURE 4:**
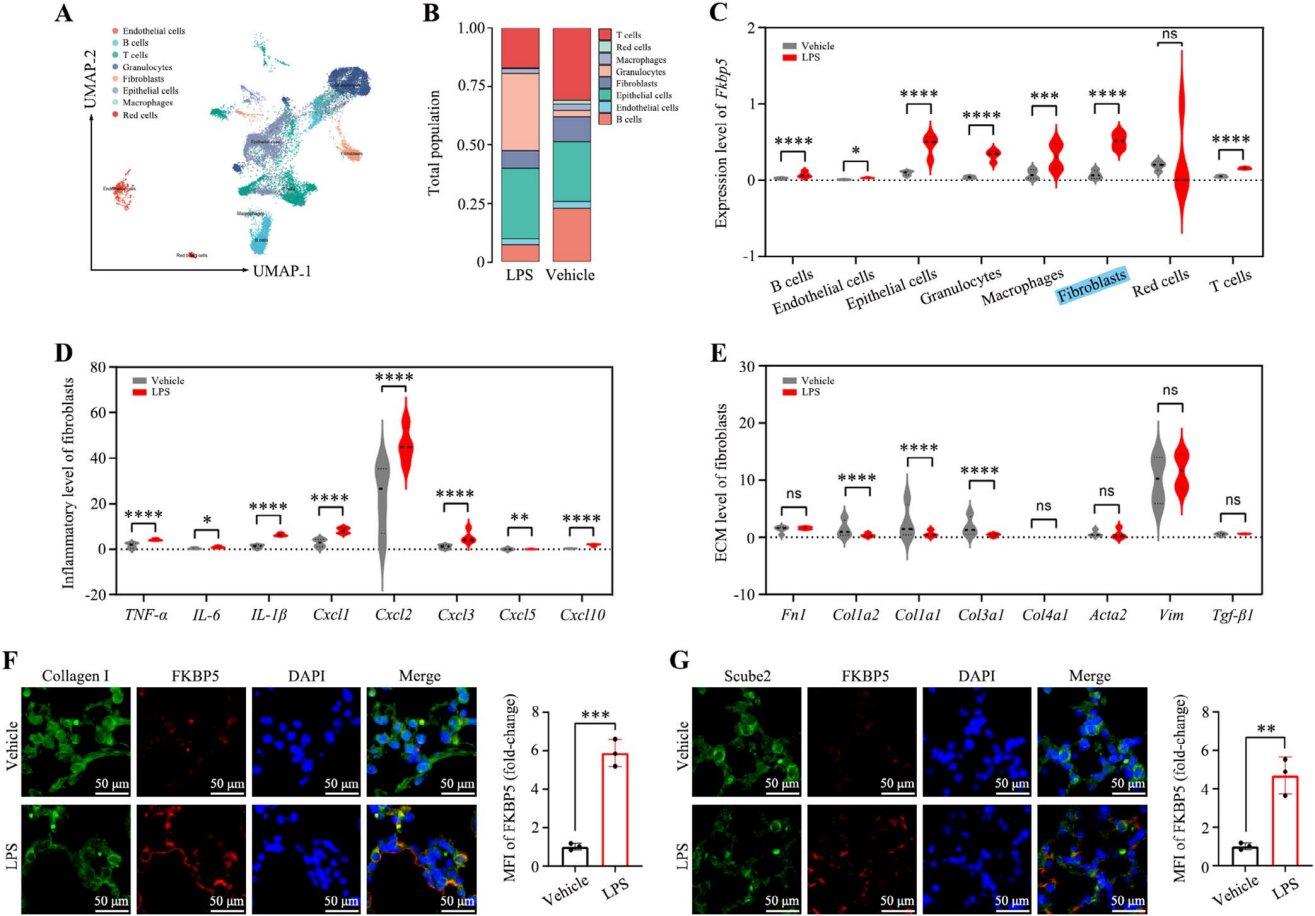
Single‐cell RNA sequencing reveals *Fkbp5* level in non‐haematopoietic cells. (A) UMAP plot showing cell clusters from lung tissue samples. (B) Cell type proportion under PBS or LPS treatment. (C) *Fkbp5* level in different cell populations under PBS or LPS. (D) Cytokine gene expression in fibroblasts under both conditions. (E) Fibrosis‐related gene expression in fibroblasts. (F) FKBP5 immunofluorescence intensity in Col1a2^+^ fibroblasts (200×; scale bar, 50 μm). (G) FKBP5 expression in Scube2^+^ alveolar fibroblasts (200×; scale bar, 50 μm). All data are presented as means ± SD from three independent experiments. *p < 0.05, **p < 0.01, ***p < 0.001, ****p < 0.0001; ns, not significant.

Single‐cell RNA sequencing revealed elevated *Fkbp5* level in fibroblasts and epithelial cells during septic ARDS (Figure 4A–C). Fibroblasts also showed increased expression of inflammatory cytokines, whereas fibrosis‐related genes were not upregulated (Figure 4D,E). Immunofluorescence further confirmed strong FKBP5 expression in alveolar fibroblasts in septic ARDS (Figure 4F,G). These findings suggest that non‐haematopoietic FKBP5, particularly in fibroblasts, plays a central role in orchestrating inflammatory responses.

### 3.4 FKBP5 Deficiency of Fibroblasts Relieved Pulmonary Inflammatory Injury in Septic ARDS Mice

**FIGURE 5:**
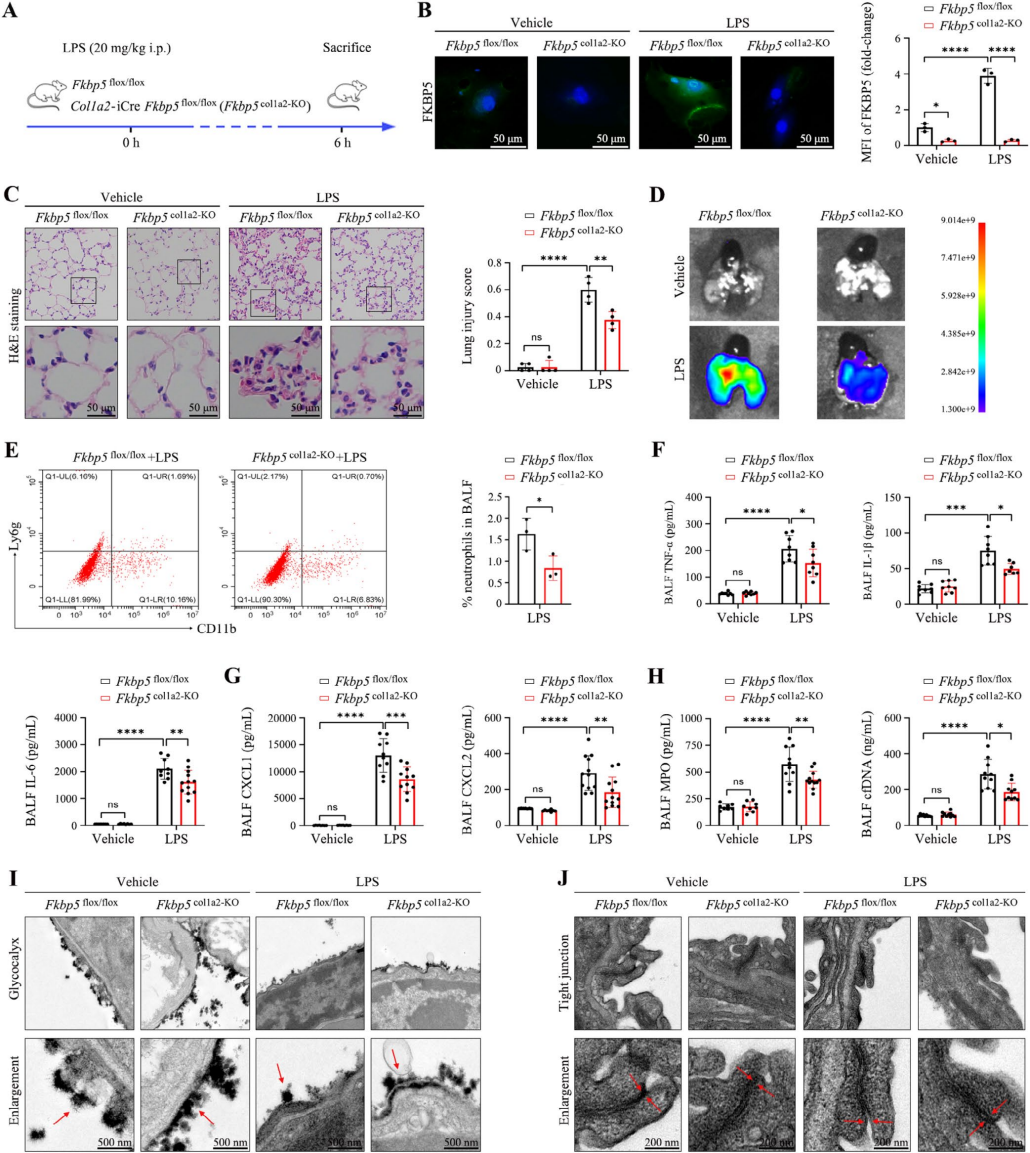
Fibroblast‐specific FKBP5 deletion reduces pulmonary inflammation in septic ARDS. (A) Septic ARDS model using *Col1a2*‐iCre *Fkbp5* ^flox/flox^ mice. (B) Immunofluorescence of FKBP5 in fibroblasts (200×; scale bar, 50 μm). (C) H&E‐stained lung sections and histopathology scoring (200×; scale bar, 50 μm). (D) FITC‐dextran assay of vascular permeability. (E) Neutrophil percentage in BALF measured by flow cytometry. (F, G) ELISA quantification of TNF‐α, IL‐1β, IL‐6, CXCL1, and CXCL2 in BALF. (H) BALF levels of MPO and cfDNA. (I) TEM of glycocalyx (8000×; scale bar, 500 nm). (J) TEM of tight junctions (20,000×; scale bar, 200 nm). All data are presented as means ± SD from three independent experiments. *p < 0.05, **p < 0.01, ***p < 0.001, ****p < 0.0001; ns, not significant.

To examine the role of fibroblast FKBP5, *Col1a2*‐iCre *Fkbp5* ^flox/flox^ mice were used to establish a fibroblast‐specific knockout model (Figure 5A,B). Histological analysis revealed that FKBP5 deletion in fibroblasts markedly reduced LPS‐induced alveolar structural damage and leukocyte infiltration (Figure 5C). FITC‐dextran assays confirmed reduced vascular leakage (Figure 5D).

Flow cytometry analysis of BALF indicated a significant reduction in neutrophil infiltration (Figure 5E). ELISA showed that FKBP5‐deficient fibroblasts significantly decreased levels of TNF‐α, IL‐1β, IL‐6, CXCL1, CXCL2, MPO, and cell‐free DNA (cfDNA) (Figure 5F–H). TEM confirmed that fibroblast FKBP5 deficiency preserved glycocalyx structure and tight junction integrity in alveolar epithelia (Figure 5I,J). These findings emphasise the critical role of fibroblast‐derived FKBP5 in mediating pulmonary inflammation.

### 3.5 Alveolar Fibroblast FKBP5 Regulates LPS‐Induced Cytokine Storms by Necroptosis

**FIGURE 6:**
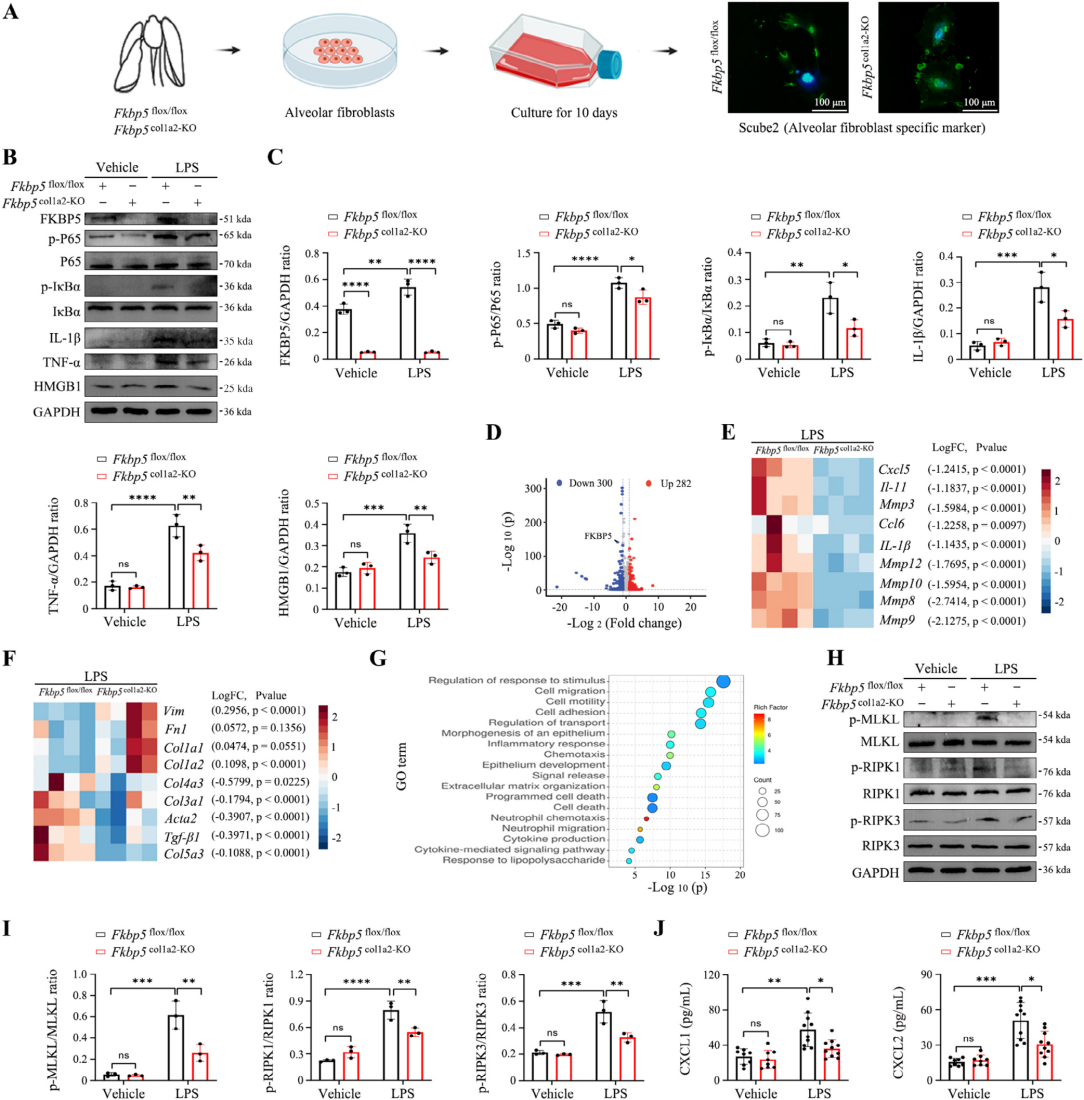
FKBP5 in mouse alveolar fibroblasts mediates LPS‐induced cytokine storms via necroptosis. (A) Schematic of enzyme‐digested fibroblast isolation and Scube2^+^ cell immunofluorescence (100×; scale bar, 100 μm). (B, C) Western blot of NF‐κB pathway proteins and cytokines (IL‐1β, TNF‐α, HMGB1) after LPS treatment (10 μg/mL, 6 h). (D) Volcano plot of differentially expressed genes (DEGs) (*p* < 0.05, fold change ≥ 2). (E, F) Heatmaps of differentially expressed proteins. (G) Gene Ontology enrichment analysis of DEGs. (H, I) Western blot validation of necroptosis activation. (J) ELISA of CXCL1 and CXCL2 levels in fibroblast supernatant. All data are presented as means ± SD from three independent experiments. *p < 0.05, **p < 0.01, ***p < 0.001, ****p < 0.0001; ns, not significant.

Mouse alveolar fibroblasts were isolated and immunofluorescence confirmed the distribution of Scube2^+^ alveolar fibroblasts (Figure 6A). After LPS stimulation (10 μg/mL, 6 h), Western blotting showed that FKBP5‐deficient fibroblasts exhibited reduced NF‐κB activation and decreased IL‐1β, TNF‐α, and HMGB1 levels (Figure 6B,C). RNA sequencing identified necroptosis as a key mechanism regulated by FKBP5 (Figure 6D–G). Western blotting confirmed reduced phosphorylation of necroptosis‐related proteins and decreased chemokine expression in FKBP5‐deficient fibroblasts (Figure 6H–J).

**FIGURE 7:**
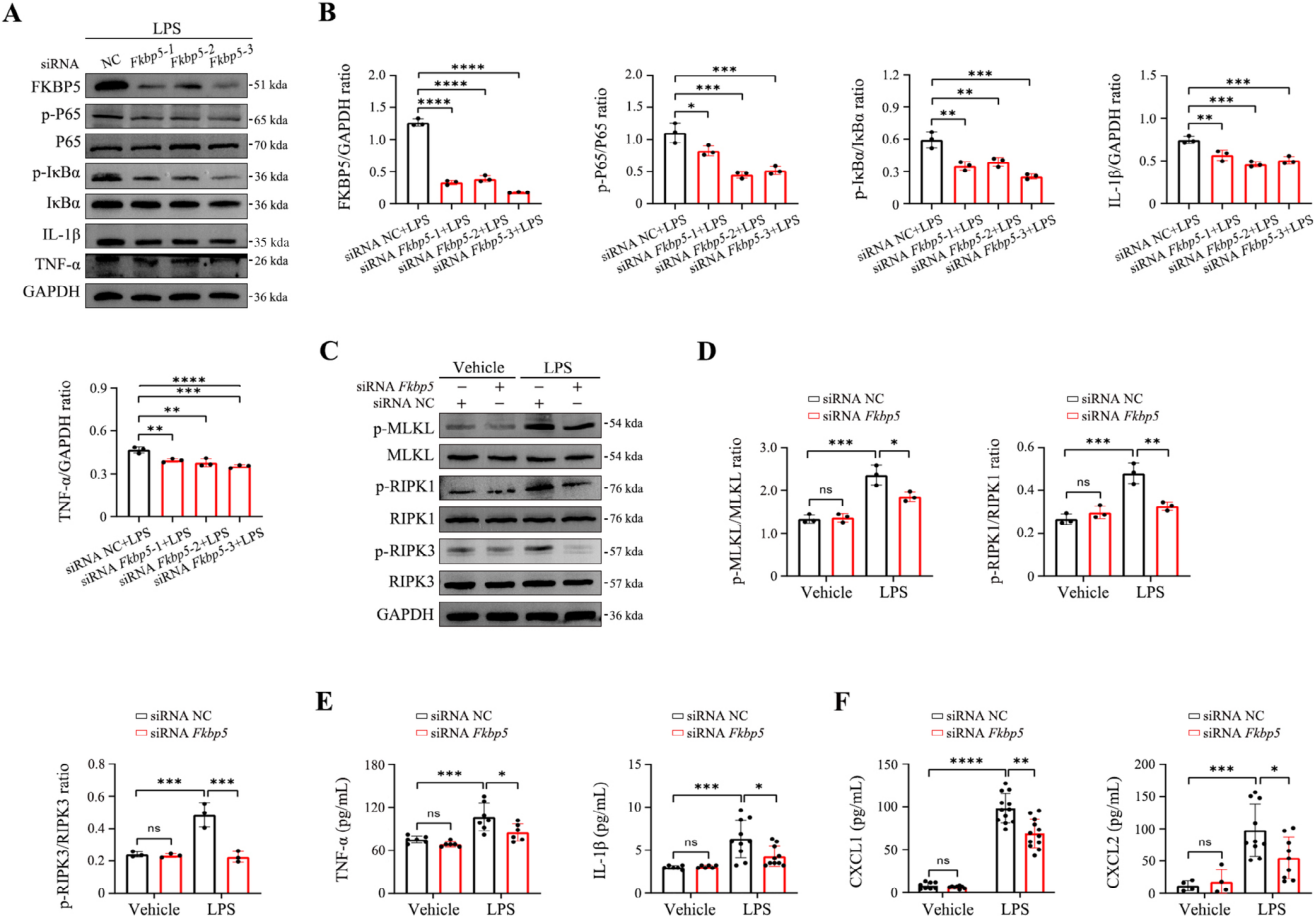
FKBP5 regulates necroptosis‐driven cytokine release in human alveolar fibroblasts. (A, B) Western blot analysis of NF‐κB signalling, IL‐1β, and TNF‐α after LPS stimulation (10 μg/mL, 6 h) with or without siRNA *Fkbp5*. (C, D) Western blot of necroptosis markers in fibroblasts with siRNA *Fkbp5*. (E, F) ELISA analysis of TNF‐α, IL‐1β, CXCL1, and CXCL2 in cell supernatant. All data are presented as means ± SD from three independent experiments. *p < 0.05, **p < 0.01, ***p < 0.001, ****p < 0.0001; ns, not significant.

Similar results were obtained in human alveolar fibroblasts (Figure 7), suggesting that FKBP5 modulates cytokine storms via necroptotic pathways.

### 3.6 FKBP5 Mediated‐Alveolar Fibroblast Necroptosis Orchestrates Inflammatory Environment in Septic ARDS

**FIGURE 8:**
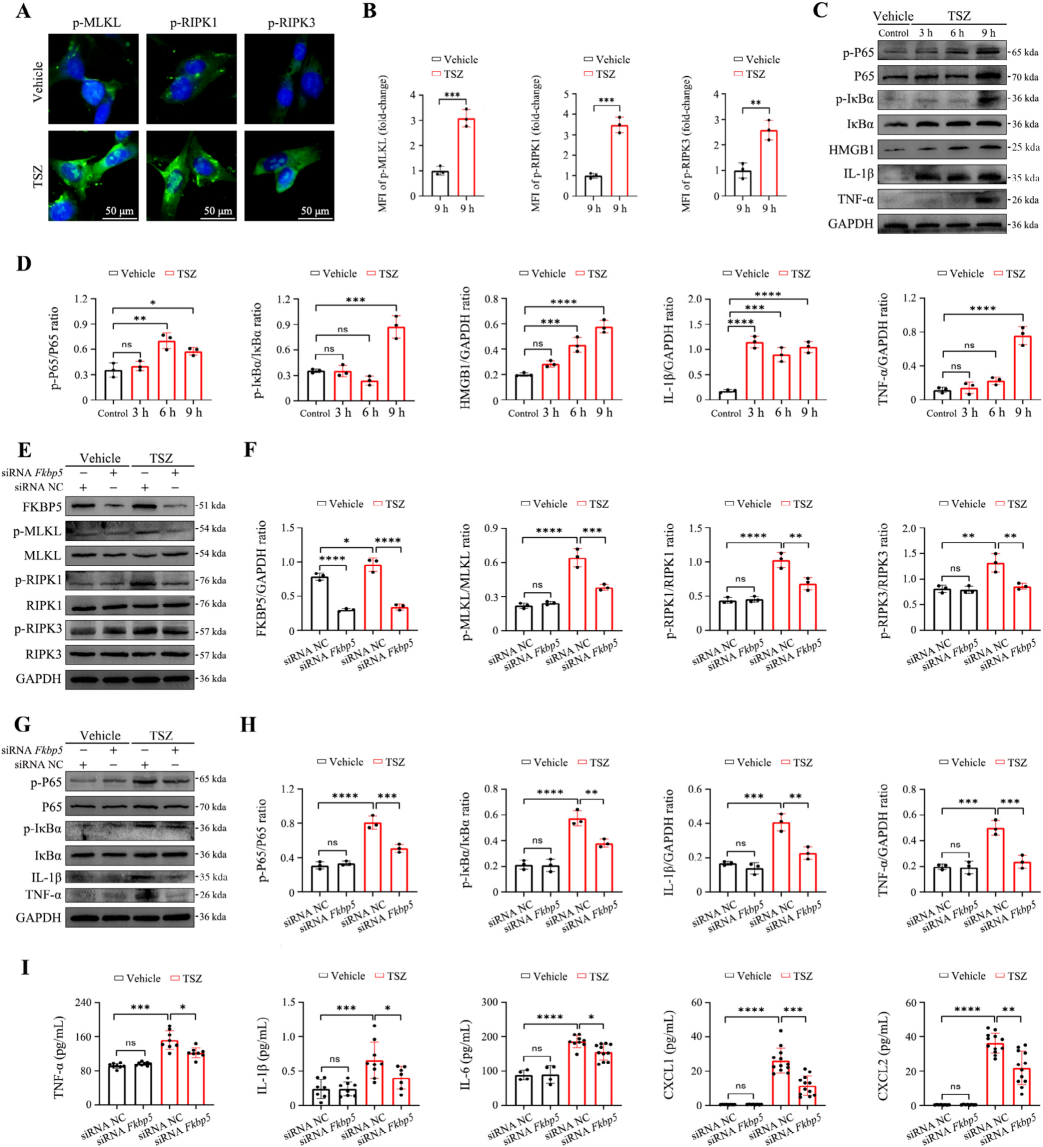
FKBP5 mediates necroptosis‐induced inflammatory signalling in human alveolar fibroblasts. (A, B) Immunofluorescence of phosphorylated RIPK1, RIPK3, and MLKL after TSZ stimulation 9 h (200×; scale bar, 50 μm). (C, D) Western blot of NF‐κB signalling, HMGB1, IL‐1β, and TNF‐α. (E, F) siRNA *Fkbp5* effect on TSZ‐induced necroptosis confirmed by Western blot. (G, H) Western blot showing suppression of NF‐κB signalling by siRNA *Fkbp5*. (I) ELISA quantification of TNF‐α, IL‐1β, IL‐6, CXCL1, and CXCL2 in fibroblast supernatants. All data are presented as means ± SD from three independent experiments. *p < 0.05, **p < 0.01, ***p < 0.001, ****p < 0.0001; ns, not significant.

SM‐164/TNF‐α combined with Z‐VAD‐FMK (TSZ) was used to establish a necroptosis model in human alveolar fibroblasts [30]. Immunofluorescence analysis revealed that TSZ significantly increased the phosphorylation levels of RIPK1, RIPK3, and MLKL, confirming successful model development (Figure 8A,B). Additionally, Western blot analysis confirmed the occurrence of an inflammatory storm following necroptosis of the alveolar fibroblasts (Figure 8C,D).

To investigate the role and molecular mechanisms of FKBP5 in necroptosis, siRNA targeting *Fkbp5* was introduced into the established necroptosis model. Western blot results demonstrated that siRNA‐mediated knockdown of *Fkbp5* significantly reduced the phosphorylation of key necroptosis proteins (Figure 8E,F). Notably, both Western blot and ELISA analyses showed that siRNA *Fkbp5* modulated necroptosis‐induced activation of the NF‐κB signalling pathway and the inflammatory response levels (Figure 8G–I).

These results underscore the pivotal role of FKBP5 in regulating necroptosis‐driven inflammatory storms in alveolar fibroblasts.

### 3.7 FKBP5 Mediates LPS‐Induced Alveolar Fibroblast Crosstalk Type II Alveolar Epithelial Cell Injury

**FIGURE 9:**
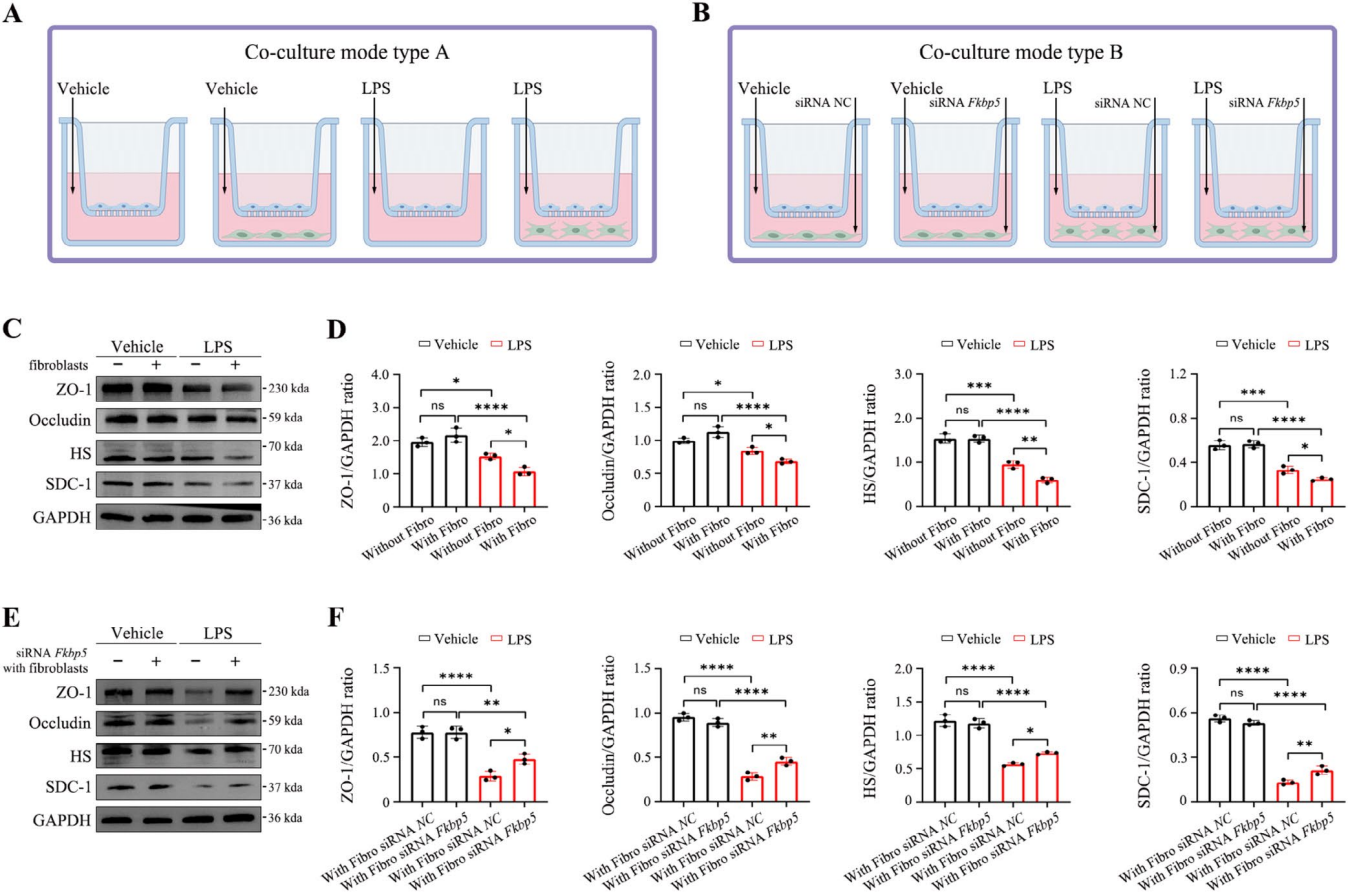
FKBP5 mediates LPS‐induced alveolar fibroblast‐epithelial crosstalk leading to type II alveolar epithelial injury. (A, B) Schematic of co‐culture model between alveolar fibroblasts and type II alveolar epithelial cells. (C, D) Expression of ZO‐1, occludin, HS, and SDC‐1 in type II epithelial cells after co‐culture with untreated or LPS‐treated alveolar fibroblasts. (E, F) Expression of epithelial barrier markers in cells co‐cultured with LPS‐treated alveolar fibroblasts transfected with siRNA *Fkbp5* versus siRNA NC. All data are presented as means ± SD from three independent experiments. *p < 0.05, **p < 0.01, ***p < 0.001, ****p < 0.0001; ns, not significant.

Given that fibroblast FKBP5 deficiency reduced glycocalyx degradation and tight junction damage, this study hypothesised that LPS‐activated fibroblasts impair type II alveolar epithelial cells. A co‐culture model was established to test this interaction (Figure 9A,B). LPS‐treated fibroblasts significantly reduced expression of glycocalyx markers (HS, SDC‐1) and tight junction proteins (ZO‐1, occludin) in type II alveolar epithelial cells (Figure 9C,D). However, co‐culture with *Fkbp5*‐silenced fibroblasts restored these markers (Figure 9E,F). These results support that FKBP5 mediates LPS‐induced alveolar fibroblast signalling that contributes to epithelial injury in septic ARDS.

## 4 Discussion

**FIGURE 10:**
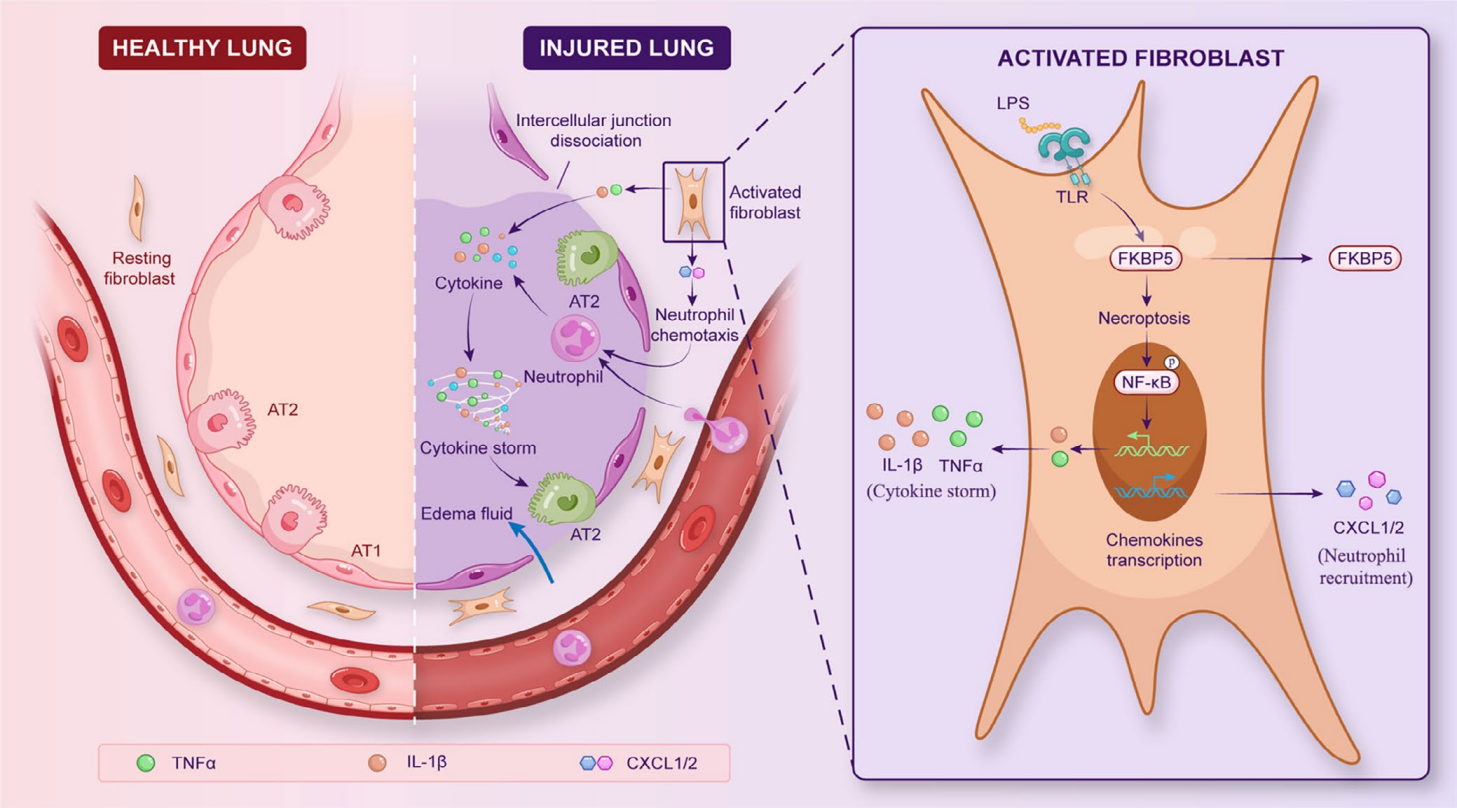
Schematic of FKBP5‐mediated alveolar fibroblast activation in septic ARDS. Diagram illustrating that FKBP5 promotes alveolar fibroblast necroptosis, leading to NF‐κB activation, cytokine release, and neutrophil recruitment, thereby shaping an inflammatory microenvironment and contributing to alveolar epithelial injury in septic ARDS.

Lung fibroblasts exhibit considerable plasticity, characterised by functional and spatial heterogeneity, which complicates efforts to define their roles. Moreover, fibroblast subtypes remain poorly characterised due to intrinsic diversity and the absence of specific markers. Tsukui et al. identified Scube2 as a selective marker of alveolar fibroblasts and documented inflammatory alterations in these cells during bleomycin‐induced pulmonary fibrosis [15]. The present study identified a Scube2^+^ alveolar fibroblast population in ARDS lesions that contributes to cytokine release and neutrophil recruitment and activation (Figure 10). These findings demonstrate a key role for alveolar fibroblasts in regulating necroptosis via FKBP5 and reveal a previously unrecognised function of this cell type in ARDS pathogenesis.

Although fibroblasts are classically regarded as mediators of tissue repair and fibrosis, emerging evidence suggests that lung fibroblasts are also responsive to inflammatory and stress‐related signals. These stimuli enable fibroblasts to acquire diverse activation states that shape the immune landscape and influence tissue damage [31]. In this study, fibroblasts within ARDS lesions exhibited a pro‐inflammatory transcriptional profile, marked by elevated chemokines and cytokines, with no corresponding increase in fibrosis‐associated markers. Isolated Scube2^+^ fibroblasts displayed early LPS‐induced phenotypes characteristic of inflammatory, rather than fibrotic, fibroblasts. These findings are consistent with previous reports implicating fibroblasts in shaping inflammatory microenvironments via cytokine and chemokine secretion [32, 33].

Necroptosis is a regulated form of necrosis, typically initiated by TNF‐α under conditions of impaired apoptosis [34]. A prior study showed that necroptosis in mouse embryonic fibroblasts promotes the transcription of pro‐inflammatory cytokines, highlighting the importance of fibroblast necroptosis in modulating tissue inflammation [30]. In the present study, transcriptomic enrichment analysis identified a strong association between FKBP5 and LPS‐induced fibroblast necroptosis. Functional assays confirmed that FKBP5 regulates this form of cell death in alveolar fibroblasts. A necroptosis‐specific model was established using TSZ to explore how FKBP5‐driven necroptosis influences the inflammatory microenvironment. Results showed that FKBP5 promotes necroptosis‐mediated release of cytokines and neutrophil chemokines.

Neutrophils play a central role in inflammatory and autoimmune disease progression [35]. Prior studies have shown that CXCL1^+^ fibroblast‐derived conditioned media prolongs neutrophil lifespan, enhances NETs formation, and contributes to persistent inflammation [36]. Neutrophils also amplify inflammation by producing IL‐1β, which activates fibroblasts, emphasising the bidirectional communication between these cell types [36]. In *Col1a2*‐iCre *Fkbp5* ^flox/flox^ mice, neutrophil accumulation and NETs formation were significantly reduced in the alveolar space following LPS exposure, suggesting that fibroblast‐derived FKBP5 influences neutrophil behaviour. In vitro studies confirmed that FKBP5 regulates the secretion of CXCL1, CXCL2, IL‐1β, and TNF‐α in LPS‐stimulated alveolar fibroblasts. These findings support a key role for fibroblast FKBP5 in modulating the inflammatory cascade of ARDS through neutrophil activation and recruitment.

The alveolar epithelial barrier is maintained by apical glycocalyx structures and intercellular tight junctions, both of which are susceptible to inflammatory disruption [37, 38]. The glycocalyx is composed primarily of SDC‐1 and HS, which are critical for barrier function and permeability regulation [39]. Tight junctions—primarily occludin and ZO‐1—facilitate intercellular signalling and selective permeability [40]. Although no direct evidence had previously supported crosstalk between alveolar fibroblasts and epithelial barrier function, the present study found that conditioned medium from LPS‐stimulated alveolar fibroblasts impaired both glycocalyx integrity and tight junction protein expression in type II alveolar epithelial cells. This effect appears to be mediated by inflammatory components in the conditioned medium, suggesting that fibroblast‐epithelial crosstalk contributes to barrier dysfunction in ARDS.

This study has limitations. Notably, functional validation using *Scube2*‐creERT2 *Fkbp5* ^flox/flox^ mice was not conducted. However, our group has generated *Scube2*‐creERT2 knock‐in mice, and future work will focus on crossing them with *Fkbp5* ^flox/flox^ mice to establish an inducible fibroblast‐specific FKBP5 knockout model for further mechanistic studies in ARDS and related diseases.

In summary, this study identified a previously unrecognised Scube2^+^ alveolar fibroblast subtype in ARDS that adopts an inflammatory phenotype early in disease progression. These fibroblasts orchestrate inflammatory signalling via FKBP5‐mediated necroptosis, promote neutrophil activation, and contribute to epithelial barrier disruption. The interplay among alveolar fibroblasts, neutrophils, and epithelial cells highlights the importance of stromal cell–immune interactions in ARDS pathogenesis and provides new mechanistic insights that may inform therapeutic strategies.

## Author Contributions

D.Z. conceived and conducted the study, performed the part of animal and cell experiments, wrote the original draft, edited, and revised the manuscript. W.L. performed the part of animal and cell experiments and analysed the RNA data. Y.X. provided substantial help with pathological evaluations. T.S. and Q.C. performed human projects and the part of cell and mouse experiments. X.H. checked and corrected the manuscript for spelling and grammar errors. X.W., Q.Q., H.W., and T.W. conceived and conducted the study. All authors contributed to the revision of the manuscript and approved the publication of the manuscript.

## Conflicts of Interest

The authors declare no conflicts of interest.

## Data Availability

The data that support the findings of this study are available from the corresponding author upon reasonable request.

## References

[1] K. D. Wick , L. B. Ware , and M. A. Matthay , “Acute Respiratory Distress Syndrome,” British Medical Journal 387 (2024): e076612. 39467606 10.1136/bmj-2023-076612

[2] M. S. Herridge , C. M. Tansey , A. Matté , et al., “Functional Disability 5 Years After Acute Respiratory Distress Syndrome,” New England Journal of Medicine 364, no. 14 (2011): 1293–1304. 21470008 10.1056/NEJMoa1011802

[3] H. Xu , S. Sheng , W. Luo , X. Xu , and Z. Zhang , “Acute Respiratory Distress Syndrome Heterogeneity and the Septic ARDS Subgroup,” Frontiers in Immunology 14 (2023): 1277161. 38035100 10.3389/fimmu.2023.1277161 PMC10682474

[4] K. Zhou , Q. Qin , and J. Lu , “Pathophysiological Mechanisms of ARDS: A Narrative Review From Molecular to Organ‐Level Perspectives,” Respiratory Research 26, no. 1 (2025): 54. 39948645 10.1186/s12931-025-03137-5 PMC11827456

[5] R. Q. Cron , G. Goyal , and W. W. Chatham , “Cytokine Storm Syndrome,” Annual Review of Medicine 74 (2023): 321–337. 10.1146/annurev-med-042921-112837 36228171

[6] C. Diorio , P. A. Shaw , E. Pequignot , et al., “Diagnostic Biomarkers to Differentiate Sepsis From Cytokine Release Syndrome in Critically Ill Children,” Blood Advances 4, no. 20 (2020): 5174–5183. 33095872 10.1182/bloodadvances.2020002592 PMC7594400

[7] S. Lin , P. Zhu , L. Jiang , et al., “Neutrophil Extracellular Traps Induced by IL‐1β Promote Endothelial Dysfunction and Aggravate Limb Ischemia,” Hypertension Research 47, no. 6 (2024): 1654–1667. 38605142 10.1038/s41440-024-01661-3

[8] D. Zhang , J. Zhang , J. Zhang , et al., “Identification of a Novel Role for TL1A/DR3 Deficiency in Acute Respiratory Distress Syndrome That Exacerbates Alveolar Epithelial Disruption,” Respiratory Research 24, no. 1 (2023): 182. 37434162 10.1186/s12931-023-02488-1 PMC10334539

[9] N. J. Meyer , L. Gattinoni , and C. S. Calfee , “Acute Respiratory Distress Syndrome,” Lancet 398 (2021): 622–637. 34217425 10.1016/S0140-6736(21)00439-6 PMC8248927

[10] D. C. Fajgenbaum and C. H. June , “Cytokine Storm,” New England Journal of Medicine 383, no. 23 (2020): 2255–2273. 33264547 10.1056/NEJMra2026131 PMC7727315

[11] N. Colás‐Algora , P. Muñoz‐Pinillos , C. Cacho‐Navas , et al., “Simultaneous Targeting of IL‐1‐Signaling and IL‐6‐Trans‐Signaling Preserves Human Pulmonary Endothelial Barrier Function During a Cytokine Storm‐Brief Report,” Arteriosclerosis, Thrombosis, and Vascular Biology 43, no. 11 (2023): 2213–2222. 37732482 10.1161/ATVBAHA.123.319695

[12] M. A. Ghonim , D. F. Boyd , T. Flerlage , and P. G. Thomas , “Pulmonary Inflammation and Fibroblast Immunoregulation: From Bench to Bedside,” Journal of Clinical Investigation 133, no. 17 (2023): e170499. 37655660 10.1172/JCI170499 PMC10471178

[13] J. M. G. Yap , T. Ueda , Y. Kanemitsu , et al., “Human Lung Fibroblasts Exhibit Induced Inflammation Memory via Increased IL6 Gene Expression and Release,” Frontiers in Immunology 13 (2022): 921728. 35941890 10.3389/fimmu.2022.921728 PMC9356221

[14] D. F. Boyd , E. K. Allen , A. G. Randolph , et al., “Exuberant Fibroblast Activity Compromises Lung Function via ADAMTS4,” Nature 587, no. 7834 (2020): 466–471. 33116313 10.1038/s41586-020-2877-5 PMC7883627

[15] T. Tsukui , P. J. Wolters , and D. Sheppard , “Alveolar Fibroblast Lineage Orchestrates Lung Inflammation and Fibrosis,” Nature 631, no. 8021 (2024): 627–634. 38987592 10.1038/s41586-024-07660-1 PMC12088911

[16] K. Ye , Z. Chen , and Y. Xu , “The Double‐Edged Functions of Necroptosis,” Cell Death & Disease 14, no. 2 (2023): 163. 36849530 10.1038/s41419-023-05691-6 PMC9969390

[17] T. Yang , C. G. Xiang , X. H. Wang , et al., “RIPK1 Inhibitor Ameliorates Pulmonary Injury by Modulating the Function of Neutrophils and Vascular Endothelial Cells,” Cell Death Discovery 10, no. 1 (2024): 152. 38521771 10.1038/s41420-024-01921-8 PMC10960796

[18] S. Xia , X. Gu , G. Wang , et al., “Regulated Cell Death of Alveolar Macrophages in Acute Lung Inflammation: Current Knowledge and Perspectives,” Journal of Inflammation Research 17 (2024): 11419–11436. 39722732 10.2147/JIR.S497775 PMC11669335

[19] G. Liccardi and A. Annibaldi , “MLKL Post‐Translational Modifications: Road Signs to Infection, Inflammation and Unknown Destinations,” Cell Death and Differentiation 30, no. 2 (2023): 269–278. 36175538 10.1038/s41418-022-01061-5 PMC9520111

[20] K. Zhang , X. Chen , R. Zhou , et al., “Inhibition of Gingival Fibroblast Necroptosis Mediated by RIPK3/MLKL Attenuates Periodontitis,” Journal of Clinical Periodontology 50, no. 9 (2023): 1264–1279. 37366309 10.1111/jcpe.13841

[21] L. L. Pelleymounter , I. Moon , J. A. Johnson , et al., “A Novel Application of Pattern Recognition for Accurate SNP and Indel Discovery From High‐Throughput Data: Targeted Resequencing of the Glucocorticoid Receptor Co‐Chaperone FKBP5 in a Caucasian Population,” Molecular Genetics and Metabolism 104, no. 4 (2011): 457–469. 21917492 10.1016/j.ymgme.2011.08.019 PMC3224211

[22] T. Bajaj , T. Ebert , L. J. Dillmann , C. Sokn , N. C. Gassen , and J. Hartmann , “SKArred 2 Death: Neuroinflammatory Breakdown of the Hippocampus,” Autophagy 20, no. 11 (2024): 2581–2583. 38934263 10.1080/15548627.2024.2373675 PMC11572194

[23] Y. L. Gan , W. J. Lin , Y. C. Fang , C. Y. Tang , Y. H. Lee , and C. J. Jeng , “FKBP51 Is Involved in LPS‐Induced Microglial Activation via NF‐κB Signaling to Mediate Neuroinflammation,” Life Sciences 351 (2024): 122867. 38914303 10.1016/j.lfs.2024.122867

[24] D. Zhang , B. Qi , Z. Peng , et al., “Heparan Sulfate Acts in Synergy With Tight Junction Through STAT3 Signaling to Maintain the Endothelial Barrier and Prevent Lung Injury Development,” International Immunopharmacology 147 (2025): 113957. 39793231 10.1016/j.intimp.2024.113957

[25] H. Wang , M. Wang , J. Chen , et al., “Interleukin‐36 Is Overexpressed in Human Sepsis and IL‐36 Receptor Deletion Aggravates Lung Injury and Mortality Through Epithelial Cells and Fibroblasts in Experimental Murine Sepsis,” Critical Care 27, no. 1 (2023): 490. 38093296 10.1186/s13054-023-04777-z PMC10717293

[26] C. Xu , L. Zhang , S. Xu , et al., “Neutrophil ALDH2 Is a New Therapeutic Target for the Effective Treatment of Sepsis‐Induced ARDS,” Cellular & Molecular Immunology 21, no. 5 (2024): 510–526. 38472357 10.1038/s41423-024-01146-w PMC11061144

[27] H. Okada , G. Takemura , K. Suzuki , et al., “Three‐Dimensional Ultrastructure of Capillary Endothelial Glycocalyx Under Normal and Experimental Endotoxemic Conditions,” Critical Care 21, no. 1 (2017): 261. 29058634 10.1186/s13054-017-1841-8 PMC5651619

[28] D. Zhang , J. Zhang , C. Xu , et al., “A Humanized Mouse Model to Study Asthmatic Airway Remodeling and Muc‐5ac Secretion via the Human IL‐33,” Allergy 79, no. 5 (2024): 1364–1367. 38226717 10.1111/all.16030

[29] B. L. Edelman and E. F. Redente , “Isolation and Characterization of Mouse Fibroblasts,” Methods in Molecular Biology 1809 (2018): 59–67. 29987782 10.1007/978-1-4939-8570-8_5

[30] K. Zhu , W. Liang , Z. Ma , et al., “Necroptosis Promotes Cell‐Autonomous Activation of Proinflammatory Cytokine Gene Expression,” Cell Death & Disease 9, no. 5 (2018): 500. 29703889 10.1038/s41419-018-0524-y PMC5923285

[31] F. Ma , O. Plazyo , A. C. Billi , et al., “Single Cell and Spatial Sequencing Define Processes by Which Keratinocytes and Fibroblasts Amplify Inflammatory Responses in Psoriasis,” Nature Communications 14, no. 1 (2023): 3455. 10.1038/s41467-023-39020-4 PMC10261041 37308489

[32] K. I. Ko , J. J. Merlet , B. P. DerGarabedian , et al., “NF‐κB Perturbation Reveals Unique Immunomodulatory Functions in Prx1+Fibroblasts That Promote Development of Atopic Dermatitis,” Science Translational Medicine 14, no. 630 (2022): eabj0324. 35108061 10.1126/scitranslmed.abj0324 PMC8979241

[33] Y. Zhang , G. Zhang , B. Dong , et al., “Pyroptosis of Pulmonary Fibroblasts and Macrophages Through NLRC4 Inflammasome Leads to Acute Respiratory Failure,” Cell Reports 44, no. 4 (2025): 115479. 40158217 10.1016/j.celrep.2025.115479 PMC12087274

[34] H. Zhou , M. Zhou , X. Liao , et al., “The Innate Immune Sensor Zbp1 Mediates Central Nervous System Inflammation Induced by *Angiostrongylus cantonensis* by Promoting Macrophage Inflammatory Phenotypes,” Advancement of Science 12, no. 11 (2025): e2413675. 10.1002/advs.202413675 PMC11923990 39853924

[35] T. Granja , D. Köhler , L. Tang , et al., “Semaphorin 7A Coordinates Neutrophil Response During Pulmonary Inflammation and Sepsis,” Blood Advances 8, no. 11 (2024): 2660–2674. 38489236 10.1182/bloodadvances.2023011778 PMC11157222

[36] X. Chen , L. OuYang , B. Qian , et al., “IL‐1β Mediated Fibroblast‐Neutrophil Crosstalk Promotes Inflammatory Environment in Skin Lesions of SLE,” Clinical Immunology 269 (2024): 110396. 39522851 10.1016/j.clim.2024.110396

[37] C. Goekeri , K. A. K. Linke , K. Hoffmann , et al., “Enzymatic Modulation of the Pulmonary Glycocalyx Enhances Susceptibility to *Streptococcus pneumoniae* ,” American Journal of Respiratory Cell and Molecular Biology 71, no. 6 (2024): 646–658. 39042016 10.1165/rcmb.2024-0003OC PMC11622634

[38] K. R. Short , J. Kasper , S. van der Aa , et al., “Influenza Virus Damages the Alveolar Barrier by Disrupting Epithelial Cell Tight Junctions,” European Respiratory Journal 47, no. 3 (2016): 954–966. 26743480 10.1183/13993003.01282-2015

[39] M. S. Kravitz , N. Kattouf , I. J. Stewart , A. A. Ginde , E. P. Schmidt , and N. I. Shapiro , “Plasma for Prevention and Treatment of Glycocalyx Degradation in Trauma and Sepsis,” Critical Care 28, no. 1 (2024): 254. 39033135 10.1186/s13054-024-05026-7 PMC11265047

[40] Z. Wang , Y. Zhong , M. Xin , et al., “Swiprosin‐1 Participates in the Berberine‐Regulated AMPK/MLCK Pathway to Attenuate Colitis‐Induced Tight Junction Damage,” Phytomedicine 135 (2024): 156111. 39369569 10.1016/j.phymed.2024.156111

